# Allergic rhinitis and periodontitis among Korean adults: results from a nationwide population-based study (2013–2015)

**DOI:** 10.1186/s12901-018-0062-3

**Published:** 2018-08-08

**Authors:** Eun-Jeong Kim, Yong-Keum Choi

**Affiliations:** 1 Department of Preventive and Social Dentistry, School of Dentistry, Seoul National University, 28 Yeongun-Dong, Jongro-Ku, Seoul, 110-749 South Korea; 2 Present Address: Department of Dental Hygiene, College of Health Science and Genome-based BioT Convergence Institute, Sun Moon University, 31460, Asan-si, Chungcheongnam-do South Korea

**Keywords:** Adults, Allergic rhinitis, Epidemiology, Periodontitis

## Abstract

**Background:**

The purpose of this study was to examine whether allergic rhinitis is associated with periodontal disease in a representative sample of elderly Korean people that was adjusted for socio-demographic factors, oral and general health behaviors, and systemic health status.

**Methods:**

A total of 10,643 subjects who were between 20 and 59 years of age participated in the Korean National Health and Nutrition Examination Survey and underwent cross-sectional examination. Medical history of allergic rhinitis was collected from participants by questionnaire; additionally, periodontal status was assessed using a Community Periodontal Index score of 3 or 4. Multivariate logistic regression analysis was conducted to adjust for socio-demographic variables, oral health status and behaviors, and general health status and behaviors. All analyses were performed using a complex sampling design.

**Results:**

Allergic rhinitis and periodontitis showed a significant inverse association. After adjusting for all confounders, a trend of decreasing periodontitis risk was observed as allergic rhinitis increased. The adjusted odds ratio of periodontitis was 0.79 (0.66–0.95) for patients with allergic rhinitis.

**Conclusion:**

A significant inverse association between allergic rhinitis and periodontal status was demonstrated in this patient population.

## Background

Periodontal disease is a major bacterial infection that causes periodontal tissue destruction and bone resorption; it may result in tooth loss [1].

Chronic periodontal disease is reported in more than 90% of the American population [2]. In Korea, the prevalence of periodontal disease in adults over 19 years of age is 29.2%; furthermore, 3 out of 10 adults have periodontal disease that requires treatment, and the number of outpatients with periodontal disease continues to increase [3].

The most significant risk factor for periodontal disease is the composition of subgingival bacteria. *Actinobacillus actinomycetemcomitans* is considered to be a pathogen implicated in several forms of aggressive periodontitis, while *Porphyromonas gingivalis* and *Tan-nerella forsythia* are considered to be important etiological agents of chronic periodontitis [4]. In addition, age, sex, stress, socioeconomic status, systemic disease, inadequate host immune response, personal oral hygiene and smoking are factors that affect the development of periodontal disease [5].

Studies on the association between periodontal and systemic diseases have been recently reported [6, 7]. The most common systemic illnesses that are associated with periodontal disease include the following: diabetes mellitus, hyperglycemia, atherosclerosis, atheromatous disease, abnormal pregnancy, respiratory disease, osteoporosis, rheumatoid arthritis, cancer, and cardiovascular disease [8]. In addition, studies have suggested that there is an association of between periodontal disease and allergic rhinitis [9].

Allergic rhinitis (AR) is a common allergic disease in which the nasal mucosa becomes hypersensitive to a variety of causative agents; it results in sinusitis, nasal congestion, otitis media, sleep disorders, and asthma [10]. AR is known to produce periodontal disease by causing chronic facial nasal obstruction and impaired breathing, leading to facial osteoarthritis, dental malocclusion, and dry mouth [11, 12].

On the other hand, the “hygiene hypothesis” suggests that infections in early infancy have preventive effects against the pathogenesis of allergies. Likewise, a low prevalence of periodontal disease has been reported in patients with allergic diseases [13].

Periodontal disease is a complex process in which pathogens interact with the host’s immune response; this process persists within the periodontal pockets and produces an acquired immune response that defends against microorganisms and pathogens [14]. Accordingly, the “hygiene hypothesis” posits that, in arthritic rhinitis and other immune diseases, exposure to various microorganisms activates and strengthens the immune system, thereby decreasing the incidence of periodontal disease. Studies investigating the association between allergic (e.g., rhinitis) and periodontal diseases are underway but are currently inadequate.

Thus, the aim of this study was to investigate the association between allergic rhinitis and periodontal disease by examining a representative sample of elderly Korean patients who were adjusted for socio-demographic factors, oral and general health behaviors, and systemic health status.

## Methods

### Study design and subject selection

The data were derived from the Sixth Korean National Health and Nutrition Examination Survey (KNHANES), which was conducted by the Korean Center for Disease Control and Prevention (KCDCP) from 2013 to 2015. The Korean Ministry of Health and Welfare performed the survey. The sampling protocol for the KNHANES was designed to employ a complex, stratified, multistage, and probability-based sampling design with proportional allocation in Korea. The KNHANES was approved by the institutional review board of the KCDC (2013-07CON-03-4C, 2013-12EXP-03-5C, and 2015–01-02-6C). The target population of the survey included all non-institutionalized civilian Korean individuals who were 1 year of age or older. The survey used stratified multistage probability sampling units that were based on geographic area, gender, and age; these sampling units were based on the households reported in the 2005 National Census Registry. The 2005 census data was used and 200 primary sampling units (PSU) were selected across Korea. The final sample for the KNHANES included 4600 households. In the KNHANES, physical and oral examinations, as well as blood sampling, were performed at mobile examination centers where trained staff members took all clinical measurements. The KNHANES included highly structured health-related questionnaires. Each participant signed an informed consent form during the survey. Previous publications of the KNHANES have described the sampling methods and survey contents in detail [15]. Out of the 22,948 participants of the KNHANES, only individuals who were between 20 and 59 years of age and had complete datasets were included in our analysis; consequently, we analyzed a total of 10,643 participants (4469 males and 6179 females) (Table 1). The following two exclusion criteria utilized: (1) participants who did not complete an oral examination and (2) subjects with one or more missing answers in their questionnaire.

**Table 1:** Association with sociodemographic characteristics, oral health behaviors, and systemic health status based on Allergic Rhinitis (*n* = 10,643)

Variables	Number	Non-AR		AR		*p* value
		N	% (95% CI)^a^	N	% (95% CI)^a^	
Age						**< 0.001**
20–29	1777	1371	77.1 (74.8–79.2)	406	22.9 (20.8–25.2)	
30–39	2643	2144	81.2 (79.4–82.8)	499	18.8 (17.2–20.6)	
40–49	2989	2556	86.2 (84.8–87.5)	433	13.8 (12.5–15.2)	
50–59	3239	2937	90.6 (89.5–91.7)	302	9.4 (8.3–10.5)	
Sex						**< 0.001**
Male	4469	3914	86.5 (85.2–87.6)	555	13.5 (12.4–14.8)	
Female	6179	5094	81.6 (80.6–82.7)	1085	18.4 (17.3–19.4)	
Monthly Household income						0.459
< 25%	905	782	86.2 (83.1–88.8)	123	13.8 (11.2–16.9)	
25–50%	2542	2153	83.8 (82.0–85.5)	389	16.2 (14.5–18.0)	
50–75%	3382	2862	84.1 (82.6–85.5)	520	15.9 (14.5–17.4)	
> 75%	3768	3167	83.7 (82.2–85.0)	601	16.3 (15.0–17.8)	
Education						**< 0.001**
Primary school	711	639	89.5 (86.5–91.9)	72	10.5 (8.1–13.5)	
Middle school	873	777	87.9 (85.1–90.2)	96	12.1 (9.8–14.9)	
High school	4063	3448	84.2 (82.9–85.5)	615	15.8 (14.5–17.1)	
College	4366	3514	80.0 (78.7–81.4)	852	20.0 (18.6–21.3)	
Smoking status						**< 0.001**
No	8314	6959	83.0 (82.0–83.9)	1355	17.0 (16.1–18.0)	
Yes	2334	2049	87.2 (85.6–88.7)	285	12.8 (11.3–14.4)	
Hypertension						**< 0.001**
No	9578	8049	83.5 (82.6–84.4)	1529	16.5 (15.6–17.4)	
Yes	1061	950	89.5 (87.2–91.4)	111	10.5 (8.6–12.8)	
Diabetes mellitus						**< 0.001**
No	10,252	8646	83.8 (83.0–84.6)	1606	16.2 (15.4–17.0)	
Yes	387	353	91.2 (87.6–93.9)	34	8.8 (6.1–12.4)	
Obesity						**< 0.001**
Underweight	500	384	76.1 (71.3–80.3)	116	23.9 (19.7–28.7)	
Normal	6756	5667	83.5 (82.5–84.5)	1089	16.5 (15.5–17.5)	
Obese	3289	2868	86.4 (85.0–87.8)	421	13.6 (12.2–15.0)	
Asthma						**< 0.001**
No	10,414	8881	84.8 (83.9–85.6)	1533	15.2 (14.4–16.1)	
Yes	234	127	54.8 (47.6–61.9)	107	45.2 (38.1–52.4)	
Atopic dermatitis						**< 0.001**
No	10,338	8811	84.7 (83.8–85.5)	1527	15.3 (14.5–16.2)	
Yes	310	197	65.2 (58.8–71.1)	113	34.8 (28.9–41.2)	
Periodontitis						**< 0.001**
No	7211	5938	81.9 (80.9–82.9)	1273	18.1 (17.1–19.1)	
Yes	2448	2201	89.6 (88.1–91.0)	247	10.4 (9.0–11.9)	
Tooth-brushing frequencies						**< 0.001**
Never	366	351	94.2 (90.5–96.6)	15	5.8 (3.4–9.5)	
Once daily	739	642	85.3 (82.1–88.0)	97	14.7 (12.0–17.9)	
Twice daily	3592	3058	84.3 (82.8–85.7)	534	15.7 (14.3–17.2)	
More than 3 times	5946	4952	83.0 (81.9–84.1)	994	17.0 (15.9–18.1)	
Use of floss						**< 0.001**
No	7917	6826	85.6 (84.6–86.5)	1091	14.4 (13.5–15.4)	
Yes	2726	2177	79.5 (77.7–81.1)	549	20.5 (18.9–22.3)	
Use of interproximal brush						**0.001**
No	8234	7027	84.8 (83.9–85.7)	1207	15.2 (14.3–16.1)	
Yes	2409	1976	81.4 (79.5–83.2)	433	18.6 (16.8–20.5)	
Use of mouthwash						
No	8407	7105	83.8 (82.8–84.8)	1302	16.2 (15.2–17.2)	0.219
Yes	2236	1898	85.1 (83.4–86.6)	338	14.9 (13.4–16.6)	
Dental visit during the past one year						0.051
No	7258	6190	84.6 (83.6–85.5)	1068	15.4 (14.5–16.4)	
Yes	3385	2813	82.9 (81.5–84.3)	572	17.1 (15.7–18.5)	

*AR* allergic rhinitis

Values are presented as number (%)

Monthly Household income: monthly average family equivalent income (−monthly average household income/ status based on Allergic Rhinitis

Obesity; Normal; Body Mass Index (BMI) 18.5 to < 25, Underweight: BMI < 18.5, and Obesity: BMI ≥25.0 kg/m^2^

Bold values denotes statistical significance at *p* < 0.05

^a^Weighted percent, 95% Confidence Interval (CI), and *p*-value obtained by Chi-square test

### Assessment of periodontitis

Trained dentists performed the oral health examination. The clinical examinations were performed with the subject seated in a dental chair while dentists utilized a light, mouth mirror, and World Health Organization (WHO) periodontal probe. The WHO community periodontal index (CPI) was used to assess the extent of periodontitis. According to the WHO guidelines, a CPI probe with a 0.5-mm ball tip was utilized with a probing force of approximately 20 g. Periodontitis was defined as a CPI greater than or equal to “code 3,” which indicates that at least one site had a pocket depth (PD) > 3.5 mm (“code 4” indicates a pocket > 5.5 mm). The index tooth numbers were 11, 16, 17, 26, 27, 31, 36, 37, 46 and 47 according to the Federation Dentaire Internationale (FDI) system. A sextant was examined only if two or more teeth were present that were not scheduled for extraction. If no index teeth were present in a sextant that was examined, all remaining teeth were probed and the highest score was recorded as the score for that sextant. The CPI was scored form 0 to 4 as follows: 0 (normal), 1 (gingivitis with bleeding on probing), 2 (presence of calculus), 3 (PD ≥ 3.5 mm) and 4 (PD ≥ 5.5 mm). The interexaminer reliability (mean of kappa value) was 0.84.

### Assessment of allergic rhinitis

AR was defined according to patients’ self-reported answers to the question, “Has your doctor ever diagnosed you with AR?” Only patients who had received at least one AR diagnosis from a certified otolaryngologist were considered.

### Assessment of potential confounders

We analyzed socio-demographic factors such as age, sex, household income, and education, as well as oral (e.g., tooth brushing frequency, prior history of periodontitis, dental visit within the past year, and the use of floss, interproximal brush, and mouthwash) and general health behaviors (e.g., smoking status and systemic illnesses such as hypertension, diabetes mellitus, obesity, asthma and atopic dermatitis). Socio-demographic, oral and general health behaviors were assessed by using the questionnaires in an interview format. General health-related factors were assessed using questionnaires, clinical examination and laboratory procedures. Monthly house hold income was categorized into quartiles and adjusted for the number of family members. Educational level was classified into the following four groups: primary school or less, middle school, high school and college. Hypertension, diabetes mellitus, asthma and atopic dermatitis by were diagnosed by a physician. Obesity was assessed using a self-administered questionnaire survey. Dentists carefully assessed each patient’s periodontal status utilizing the Community Periodontal Index of Treatment Needs (CPITN) to quantify the extent of periodontitis. The selected index tooth numbers were 11, 16, 17, 26, 27, 31, 36, 37, 46 and 47. The CPITN Index was scored from 0 to 4 as follows: 0 (normal), 1 (gingivitis with bleeding on probing), 2 (presence of calculus), 3 (pocket depth ≥ 3.5 mm) and 4 (pocket depth ≥ 5.5 mm). Periodontal status was grouped into the following two categories: absence (CPI of 1 to 2) and presence (CPI of 3 to 4) of periodontitis. The frequency of tooth brushing was divided into the following four groups: never, once daily, twice daily and more than three times daily. The use of floss, interproximal brush and mouthwash, as well as dental visits during the past year, were grouped into binomial yes or no categories.

### Statistical analysis

The complex sampling design of the survey was utilized to obtain the variances and individual weight of each analyzed factor. To investigate the characteristics of AR and periodontitis patients, a chi-squared test of complex sample analysis with weight application was performed to estimate the weighted proportions (95% confidence interval [CI]) of the total sample population. Multivariate logistic regression analyses were sequentially applied to assess the association between AR and other variables after adjusting for age, sex, household income, education, smoking status, hypertension, diabetes mellitus, asthma, atopic dermatitis, tooth brushing frequency, dental visit during the past year, and the use of floss, interproximal brush, and mouthwash.

A multiple logistic regression analysis of complex samples was carried out to evaluate the weight-adjusted association between AR and periodontitis. Model 1 represents a crude association. Model 2 is adjusted for sociodemographic factors. Model 3 is adjusted for all the variables in Model 2, including oral health status and behaviors. Model 4 is adjusted for all the variables in Model 3, including general health status and behaviors.

To enable the complex survey design involving stratified, random and cluster sampling, the SPSS Complex Samples Procedures were used for all statistical analyses (IBM SPSS Statistics Version 21, IBM Inc., Chicago, IL, USA). A *p*-value < 0.05 was considered statistically significant.

## Results

### Characteristics of the study participants

The characteristics of the study participants were categorized by AR. Of the 10,643 participants that were included in the analyses, 1640 (15.4%) had AR (Table 1). In our analysis, chi-squared tests were used to investigate the univariate association between confounding variables and AR. AR was more prevalent among younger participants, females, participants with higher education, and non-smokers. Among individuals with AR, asthma, atopic dermatitis, hypertension, obesity and periodontitis were observed to be more prevalent. Participants with AR were also observed to have greater tooth-brushing frequency and an increased use of both floss and interproximal brushes.

### Association between periodontitis and potential confounding variables

The characteristics of the study subjects were categorized by periodontal status after controlling for all possible confounders (Table 2). The demographic characteristics of the participants were noteworthy for a higher percentage of males with periodontitis (30.5%) compared to females (18.7%); the odds ratio (OR) was 1.44 (95% CI: 1.27–1.63) for males with periodontitis. Periodontitis was present in 26.5% of the non-AR group and 15.8% of the AR group; the crude OR was 1.53 (95% CI: 1.28–1.82) for participants with both AR and periodontitis.

**Table 2:** Univariate association with sociodemographic characteristics, oral health and general health behaviors, and systemic health status based on periodontitis (*n* = 9659)

Variables	Number	Periodontitis				OR (95% CI)
		No		Yes		
		N	% (95% CI)^a^	N	% (95% CI)^a^	
Age						
20–29	1657	1582	95.3 (93.9–96.4)	75	4.7 (3.6–6.1)	0.07 (0.05–0.09)
30–39	2497	2128	84.4 (82.1–86.4)	369	15.6 (13.6–17.9)	0.27 (0.22–0.33)
40–49	2774	1978	69.4 (67.1–71.6)	796	30.6 (28.4–32.9)	0.61 (0.53–0.70)
50–59	2982	1712	55.2 (52.7–57.6)	1270	44.8 (42.4–47.3)	Reference
Sex						
Male	4191	2819	69.5 (67.6–71.3)	1372	30.5 (28.7–32.4)	1.44 (1.27–1.63)
Female	5719	4581	81.3 (79.9–82.7)	1138	18.7 (17.3–20.1)	Reference
Monthly Household income						
< 25%	853	562	66.1 (62.0–70.1)	291	33.9 (29.9–38.0)	1.08 (0.86–1.37)
25–50%	2357	1700	73.4 (71.1–75.6)	657	26.6 (24.4–28.9)	0.99 (0.84–1.18)
50–75%	3136	2379	76.3 (74.2–78.2)	757	23.7 (21.8–25.8)	0.92 (0.78–1.08)
> 75%	3516	2724	78.0 (75.9–79.9)	792	22.0 (20.1–24.1)	Reference
Education						
Primary school	639	355	54.9 (49.8–59.9)	284	45.1 (40.1–50.2)	2.76 (2.13–3.57)
Middle school	801	474	57.7 (53.8–61.5)	327	42.3 (38.5–46.2)	2.82 (2.30–3.47)
High school	3728	2781	75.5 (73.6–77.2)	947	24.5 (22.8–26.4)	1.34 (1.16–1.54)
College	4036	3291	81.5 (79.7–83.2)	745	18.5 (16.8–20.3)	Reference
Smoking status						
No	2084	1301	64.2 (61.5–66.8)	783	35.8 (33.2–38.5)	1.69 (1.47–1.95)
Yes	7575	5910	79.0 (77.5–80.3)	1665	21.0 (19.7–22.5)	Reference
Hypertension						
No	8931	6837	77.2 (75.8–78.6)	2094	22.8 (21.4–24.2)	0.56 (0.46–0.68)
Yes	979	563	55.8 (51.9–59.7)	416	44.2 (40.3–48.1)	Reference
Diabetes mellitus						
No	9557	7221	76.2 (74.8–77.6)	2336	23.8 (22.4–25.2)	0.53 (0.40–0.71)
Yes	353	179	49.4 (43.9–55.0)	174	50.6 (45.0–56.1)	Reference
Obesity						
Underweight	454	393	86.9 (83.0–90.0)	61	13.1 (10.0–17.0)	0.45 (0.32–0.64)
Normal	6282	4858	77.9 (76.3–79.4)	1424	22.1 (20.6–23.7)	0.75 (0.66–0.85)
Obese	3088	2069	68.0 (65.8–70.1)	1019	32.0 (29.9–34.2)	Reference
Asthma						
No	9438	7035	75.2 (73.7–76.6)	2403	24.8 (23.4–26.3)	1.14 (0.77–1.69)
Yes	221	176	79.7 (72.8–85.2)	45	20.3 (14.8–27.2)	Reference
Atopic dermatitis						
No	9365	6946	74.8 (73.3–76.2)	2419	25.2 (23.8–26.7)	2.58 (1.58–4.22)
Yes	294	265	90.2 (85.1–93.7)	29	9.8 (6.3–14.9)	Reference
Allergic rhinitis						
No	8139	5938	73.5 (72.0–75.0)	2201	26.5 (25.0–28.0)	1.53 (1.28–1.82)
Yes	1520	1273	84.2 (81.8–86.3)	247	15.8 (13.7–18.2)	Reference
Tooth-brushing frequencies						
Never	270	184	66.7 (59.4–73.3)	86	33.3 (26.7–40.6)	1.16 (0.71–1.88)
Once daily	670	418	63.3 (58.9–67.6)	252	36.7 (32.4–41.1)	1.36 (1.10–1.67)
Twice daily	3283	2377	72.8 (70.7–74.7)	906	27.2 (25.3–29.3)	1.11 (0.99–1.23)
1 (0.99–1.23)re	5433	4230	78.9 (77.3–80.4)	1203	21.1 (19.6–22.7)	Reference
Use of floss						
No	7136	5073	71.9 (70.2–73.5)	2063	28.1 (26.5–29.8)	1.70 (1.48–1.97)
Yes	2520	2136	85.3 (83.5–86.9)	384	14.7 (13.1–16.5)	Reference
Use of interproximal brush						
No	7448	5522	74.8 (73.3–76.3)	1926	25.2 (23.7–26.7)	0.92 (0.78–1.07)
Yes	2208	1687	76.9 (74.2–79.3)	521	23.1 (20.7–25.8)	Reference
Use of mouthwash						
No	7602	5701	75.8 (74.3–77.3)	1901	24.2 (22.7–25.7)	0.73 (0.63–0.84)
Yes	2054	1508	73.2 (70.6–75.6)	546	26.8 (24.4–29.4)	Reference
Dental visit during the past one year						
No	6563	4878	75.1 (73.5–76.6)	1685	24.9 (23.4–26.5)	0.88 (0.78–1.00)
Yes	3093	2331	75.7 (73.6–77.7)	762	24.3 (22.3–26.4)	Reference

AR, allergic rhinitis

Values are presented as number (%)

Monthly Household income: monthly average family equivalent income (−monthly average household income/behaviors, and systemic health stat

Obesity; Normal; Body Mass Index (BMI) 18.5 to < 25, Underweight: BMI < 18.5, and Obesity: BMI ≥25.0 kg/m^2^

Bold values denotes statistical significance at *p* < 0.05

^a^Weighted percent, 95% Confidence Interval (CI), and *p*-value obtained by Chi-square test

### Association between AR and periodontitis

The associations between periodontitis and AR was analyzed using multivariable logistic regression models. These analyses revealed that the AR was inversely associated with the risk for periodontitis throughout the adjustment processes utilized in Models 1, 2, 3, and 4 (Table 3). After adjusting for socio-demographic factors, oral and general health behaviors, and general health factors, the inverse associations between AR and periodontitis remained significant.

**Table 3:** Adjusted odds rations (OR) and 95% confidence intervals (CI) between the periodontitis and allergic rhinitis in multiple models (9659)

Variable	Number	OR (95% CI)			
		Model 1^a^	Model 2^b^	Model 3^c^	Model 4^d^
Allergic rhinitis					
Yes	2448	**0.52 (0.44–0.62)**	**0.74 (0.62–0.89)**	**0.76 (0.63–0.91)**	**0.79 (0.66–0.95)**
No	7211	1	1	1	1

*OR* odds ratio, *CI* confidence interval

Bold values denotes statistical significance at *p* < 0.05

^a^Model 1 was unadjusted association

^b^Model 2 was adjusted for sex, age, household income and education

^d^Model 3 was adjusted for all variables in model 2 and use of floss, use of interproximal brush, use of mouthwash, tooth-brushing frenquencies and dental visit during the past one year

^d^Model 4 was adjusted for all variables in model 3 and smoking status, hypertension, diabete mellitus, obesity, asthma and atopic dermatitis

## Discussion

Our results demonstrated that AR is inversely associated with periodontitis among Korean adults (aged 20–59 years) and that this association remained significant even after adjusting for age, sex, household income, smoking, hypertension, diabetes mellitus, obesity, asthma, atopic dermatitis, periodontitis, tooth brushing frequency, dental visits during the past year, and the use of floss, interproximal brush, and mouthwash. These results were based on a national Korean population sample consisting of 10,643 participants and supported evidence from previous studies showing an inverse association between allergic disease and periodontitis [13]. To the best of our knowledge, this study provides the first evidence showing that AR is inversely associated with the prevalence of periodontitis in middle-aged adults.

We found that AR was more prevalent in women compared to men, which is similar to the results of another study conducted in the US [16]. Our results also showed that participants with lower age had a higher prevalence of AR; accordingly, Skoner reported that AR symptoms develop before the age of 20 years in approximately 80% of cases [17]. In addition, participants who had allergic diseases, such as asthma and atopic dermatitis, showed a higher prevalence of AR. Once a patient has become sensitized to allergens, subsequent exposures appears to trigger a cascade of events that result in AR symptoms [17].

There were a few studies on allergic disease and periodontitis in the literature. Friedrich et al. [13] conducted a study in a large Germany population sample to investigate whether allergic diseases such as hayfever, house dust mite allergy, and asthma were associated with periodontal disease; their study found that an inverse association between periodontitis and respiratory allergies. The present study, also showed an inverse association between periodontitis and allergic rhinitis as well as other allergic disease such as asthma and atopic dermatitis.

Several biological mechanisms have been suggested to explain how periodontopathic bacteria influence allergic rhinitis. Periodontal disease represents early infection, which may elicit an immune response, and the primitive conditions for the onset of periodontal disease may emerge in childhood [17]. Strachan [18] posited the “hygiene hypothesis,” which provides a potential explanation for the T-helper type 1 (Th1)/T-helper type 2 (Th2) paradigm [19, 20]. Allergic disease is Th2-mediated and characterized by the release of IgE; however, bacterial and viral infections are more likely to be Th1-mediated. Th2-derived cytokines inhibit the development of Th1 cells and vice versa. The infection-induced Th1-specific cytokine inhibits the development of allergen-specific Th2 cells. Several studies also supported the hygiene hypothesis [21, 22], suggesting that bacterial colonization elicit a systemic reaction that prevents the onset of allergic diseases.

Little is known about possible relationship between rhinitis and periodontitis. One study by Hung et al. has reported that rhinitis is associated with periodontitis, which is a finding that is different from the current study [9]. It should be noted that Hung et al. did not fully adjust for systemic factors. And another study by Choi et al. has also reported that asthma diagnosis is associated with tooth loss, however, this study focused on asthma not on allergic rhinitis [23].

There are four major strengths of our study. First, the KNHANES data utilized for this study was a large national survey that is representative of the Korean population. Seconds, professional examiners documented the general health examination and laboratory analyses. Third, trained dentists performed periodontal examinations and assessed oral health status. These results provide the first evidence linking periodontitis and AR among middle-aged adults. Finally, confounding factors showed a statistically significant independent association with periodontitis in our data, thus corroborating the reliability of our data.

This study has some limitations. Because the KNHANES is a cross-sectional survey, it is not possible to identify a causal relationship between AR and periodontitis; however, the biological plausibility of this association may indicate this relationship. Furthermore, the periodontal status was assessed using the CPI; therefore, the prevalence of periodontitis may have been overestimated or underestimated because the CPI’s use of representative teeth may include pseudo pockets [24]. Additionally, the method for defining AR was self-questionnaire, which runs the risk of recall bias, even if the question involves a physician’s diagnosis. Case control and cohort studies are needed to investigate the relationship between periodontal disease and AR.

Notwithstanding these limitations, the results of our study are reliable enough to test the hypothesis that AR is inversely associated with periodontitis.

## Conclusion

In conclusion, this study found an inverse association between AR. Further case control and cohort studies are needed to confirm the hypothesis and mechanism of this inverse association between AR and periodontitis.

## Abbreviations

AR: Allergic rhinitis
CI: Confidence interval
CPI: Community periodontal index
CPITN: Community Periodontal Index of Treatment Needs
FDI: Federation Dentaire Internationale
KCDCP: Korean Center for Disease Control and Prevention
KNHANES: Korean National Health and Nutrition Examination Survey
OR: Odds ratio
PD: Pocket depth
PSU: Primary sampling units
Th1: T-helper type 1
Th2: T-helper type 2
WHO: World Health Organization
